# Reproducibility and Repeatability of CBCT-Derived Radiomics Features

**DOI:** 10.3389/fonc.2021.773512

**Published:** 2021-11-17

**Authors:** Hao Wang, Yongkang Zhou, Xiao Wang, Yin Zhang, Chi Ma, Bo Liu, Qing Kong, Ning Yue, Zhiyong Xu, Ke Nie

**Affiliations:** ^1^ Department of Radiation Oncology, Shanghai Chest Hospital, Shanghai Jiaotong University, Shanghai, China; ^2^ Department of Radiation Oncology, Rutgers Cancer Institute of New Jersey, Rutgers-Robert Wood Johnson Medical School, New Brunswick, NJ, United States; ^3^ Institute of Modern Physics, Fudan University, Shanghai, China; ^4^ Department of Radiation Oncology, Zhongshan Hospital, Fudan University, Shanghai, China

**Keywords:** reproducibility, repeatability, longitudinal CBCT radiomics, imaging protocol, in-treatment image

## Abstract

**Purpose:**

This study was conducted in order to determine the reproducibility and repeatability of cone-beam computed tomography (CBCT) radiomics features.

**Methods:**

The first-, second-, and fifth-day CBCT images from 10 head and neck (H&N) cancer patients and 10 pelvic cancer patients were retrospectively collected for this study. Eighteen common radiomics features were extracted from the longitudinal CBCT images using two radiomics packages. The reproducibility of CBCT-derived radiomics features was assessed using the first-day image as input and compared across the two software packages. The site-specific intraclass correlation coefficient (ICC) was used to quantitatively assess the agreement between packages. The repeatability of CBCT-based radiomics features was evaluated by comparing the following days of CBCT to the first-day image and quantified using site-specific concordance correlation coefficient (CCC). Furthermore, the correlation with volume for all the features was assessed with linear regression and *R* ^2^ as correlation parameters.

**Results:**

The first-order histogram-based features such as skewness and entropy showed good agreement computed in either software package (ICCs ≥ 0.80), while the kurtosis measurements were consistent in H&N patients between the two software tools but not in pelvic cases. The ICCs for GLCM-based features showed good agreement (ICCs ≥ 0.80) between packages in both H&N and pelvic groups except for the GLCM-correction. The GLRLM-based texture features were overall less consistent as calculated by the two different software packages compared with the GLCM-based features. The CCC values of all first-order and second-order GLCM features (except GLCM-energy) were all above 0.80 from the 2-day part test–retest set, while the CCC values all dropped below the cutoff after 5-day treatment scans. All first-order histogram-based and GLCM-texture-based features were not highly correlated with volume, while two GLRLM features, in both H&N and pelvic cohorts, showed *R* ^2^ ≥0.8, meaning a high correlation with volume.

**Conclusion:**

The reproducibility and repeatability of CBCT-based radiomics features were assessed and compared for the first time on both H&N and pelvic sites. There were overlaps of stable features in both disease sites, yet the overall stability of radiomics features may be disease-/protocol-specific and a function of time between scans.

## Introduction

Radiomics, the high-throughput mining of image features from routine medical images, provides a quantitative and robust method to assess tumor heterogeneity. It can serve as a powerful tool for precision medicine in cancer treatment. However, the current work primarily focuses on diagnostic metrics which neglects the treatment effect. The involvement of in-treatment image should be investigated for a direct estimation of treatment outcome.

Daily cone-beam computed tomography (CBCT) images are originally developed for patient setup and have accordingly been acquired with low imaging dose. The images have substantially more scatter than diagnostic CT due to the flat-panel detector design. However, in many cases, these images are acquired at every fraction of treatment during the whole course of radiation therapy and may function as a timely biomarker for treatment-induced changes (1–4). Benjamin et al. (1) reported that serial changes from CBCT images during head and neck (H&N) radiotherapy can improve chronic xerostomia prediction. Hebert et al. (5) revealed that intersite heterogeneity captured from CBCT could predict outcomes in patients with high-grade serous ovarian cancer. Despite the promising potential of radiomics features from longitudinal CBCT images, the stability of these features should be addressed.

In addition, given the increasing number of radiomics-based studies, investigators have built many in-house software packages, and several radiomics platforms are available for public use. The inherent variations from algorithm implementation, image preprocessing, and mathematical definitions could cause large differences in radiomics feature computation. Moreover, the differences in disease-specific image settings such as mAs, kVp, and image resolution could also contribute to computational variations. The lack of understanding of stability has slowed the clinical implementation of many promising radiomics-based diagnosis or prognosis schemes. One can be guided on the use of radiomics features derived from CBCT images in clinical studies only after the sources of variations are understood.

As such, in this work, we evaluated the reproducibility and repeatability of radiomics features derived from longitudinal CBCT images for two distinct clinical sites. The agreement of these features across two commercially available platforms was also analyzed.

## Methods and Materials

### Medical Imaging Data

Ten H&N cancer patients and 10 pelvic cancer patients were randomly selected retrospectively. The study was approved by the institutional review board. All CBCT patient images were acquired on Varian Truebeam On-Board Imager (OBI). The images for selected cases were obtained using the exact same imaging protocol for that particular disease site and the same machine to minimize variations from the imaging settings. The imaging system was maintained by qualified medical physicists and service engineers at least on a monthly basis in terms of image contrast, resolution, distortion, and Hounsfield unit (HU) consistency. The treatment machine along with the imaging system has been credentialed for clinical trials (including head and neck and pelvis) with IROC phantoms. For each patient, the first-, second-, and fifth-day CBCT images were collected. All CBCT patient images were acquired on the Varian Truebeam OBI. The H&N images were taken with a peak tube voltage of 100 kVp and tube current of 150 mAs. Images were reconstructed with 512 × 512 grid and pixel dimensions of 0.511 × 0.511 × 2 mm slice thickness. The pelvic images were taken under the protocol of 125 kVp and 100 mAs and reconstructed to 512 × 512 grid size with a pixel resolution of 0.908 × 0.908 × 2 mm slice thickness.

**Figure 1:**
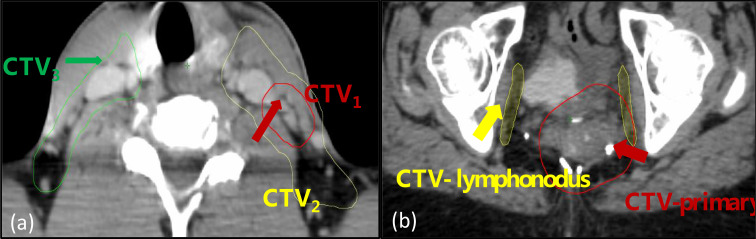
The regions-of-interest (ROIs) included in the analysis: **(A)** a head and neck case with three volumes designated as CTV_1_, CTV_2_, and CTV_3_; **(B)** a pelvic case with two volumes designated as CTV-primary and CTV-lymphonodus.

For each of the patients, the contours from the treatment planning CT were transformed to the CBCT image sets using Velocity (Varian, Palo Alto, USA) with multipass deformable registration. For each CBCT image set, the clinical target volumes (CTVs), three for H&N and two for pelvic as shown in **Figure 1**, were selected for radiomics analysis.

### Radiomics Feature Extraction

Two widely used open-source radiomics packages—IBEX v1.0 beta (The University of Texas MD Anderson Cancer Center) and LIFEx v5.10 (https://www.lifexsoft.org/)—were used for comparison (6–11). Each radiomics package was capable of calculating various types of radiomics features including first-order histogram features, second-order gray-level co-occurrence matrix (GLCM) features, and gray-level run length matrix (GLRLM) features. In this study, only those features with the same mathematical definitions were selected. The common features are shown in **Table 1**. All the features were calculated for each CTV from every CBCT fraction with both software tools, respectively. To eliminate variation from image preprocessing, no additional and only the default preprocessing was applied. CBCT image parameters such as pixel size and gray levels number were set at the same value, and differences in algorithm implementation were reduced to allow for the greatest possible consistency check between packages.

**Table 1:** First- and second-order radiomics features shared the same definition in the two packages.

First-order histogram features	Skewness, kurtosis, entropy
Second-order GLCM features	Energy, jointEntropy, dissimilarity, correction
Second-order GLRLM features	Short-run emphasis (SRE), long-run emphasis (LRE), gray-level non-uniformity (GLN), run length non-uniformity (RLN), low gray-level run emphasis (LGLRE), high gray-level run emphasis (HGLRE), short-run low gray-level emphasis (SRLGLE), short-run high gray-level emphasis (SRHGLE), long-run low gray-level emphasis (LRLGLE), long-run high gray-level emphasis (LRHGLE), run percentage (RP)

### Reproducibility and Repeatability of Radiomics Features

The reproducibility was assessed using the first-day CBCT images (with 30 H&N CTVs and 20 pelvic CTVs) as input for each of the two radiomics packages. A total of 18 common features, consisting of 3 first-order histogram-based features and 15 second-order texture-based features (4 from GLCM and 11 from GLRLM), were compared. The agreements between software packages were examined by qualitatively comparing distribution through boxplots. The intraclass correlation coefficient (ICC) was further used to quantitatively assess the agreements between packages. It compares the variability across software packages *vs*. the variability across patients. The two clinical sites have different CBCT imaging protocols as the H&N images have less scatter, smaller field of view, and higher image resolution compared with pelvic cases. The robust features found in the H&N protocol may not be extendable to images with the pelvic protocol. The reproducibility of radiomics features in terms of different imaging protocols was evaluated with site-specific ICCs. The ICC values were stratified to indicate “good” (ICC ≥ 0.8), “moderate” (0.8 > ICC ≥ 0.5), or “poor” (ICC < 0.5) agreement (12–15).

Repeatability was assessed using longitudinal CBCT images as test–retest datasets. The radiomics features derived from first-day CBCT images were used as the baselines, and the second- and the fifth-day images were compared with the baseline. The concordance correlation coefficient (CCC) was used to examine agreement between radiomics features derived from the test–retest scans. Site-specific CCCs were evaluated specifically. The cutoff value was chosen based on the recommended criteria by McBride et al. (16) that a correlation of 0.8 reflects good strength-of-agreement; otherwise, it is poor. Furthermore, the correlation with volume for all the features was assessed with linear regression and *R* ^2^ as correlation parameters. Statistical analysis was performed using the package psych in R (version 3.2.3).

## Results

**Figure 2:**
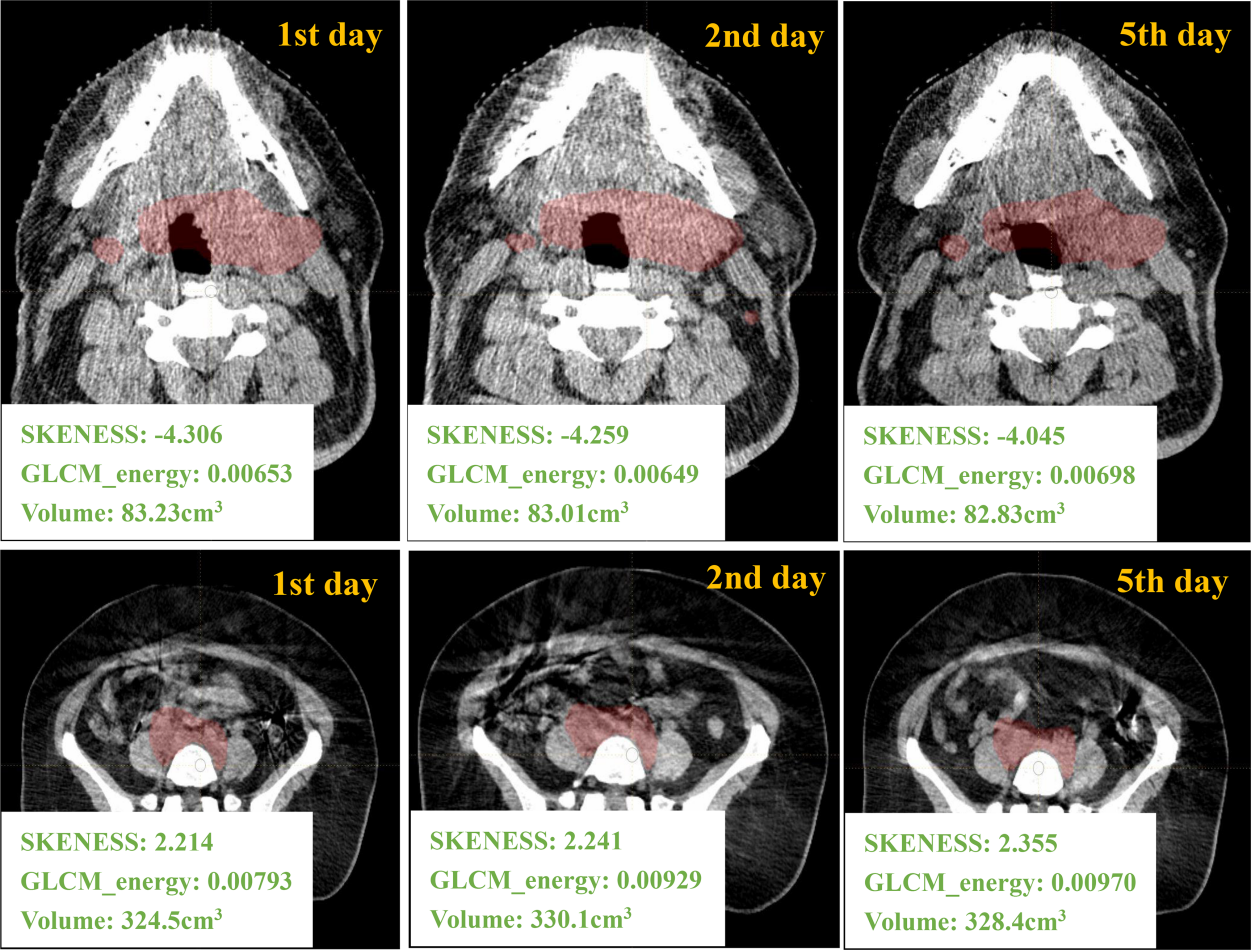
Different days of CBCT images and the corresponding radiomics/volume values from one H&N ROI and one pelvic ROI.

**Figure 2** gives an example of a H&N case and a pelvic case with different-day CBCT images. The values of first-order feature such as histogram_skewness and of second-order feature such as GLCM_energy from LIFEx package are shown. Histogram_skewness which focused on total intensity distribution was very similar across days, but GLCM_energy which focused on internal heterogeneity showed variations.

### Reproducibility of CBCT-Based Radiomics Features

**Figure 3:**
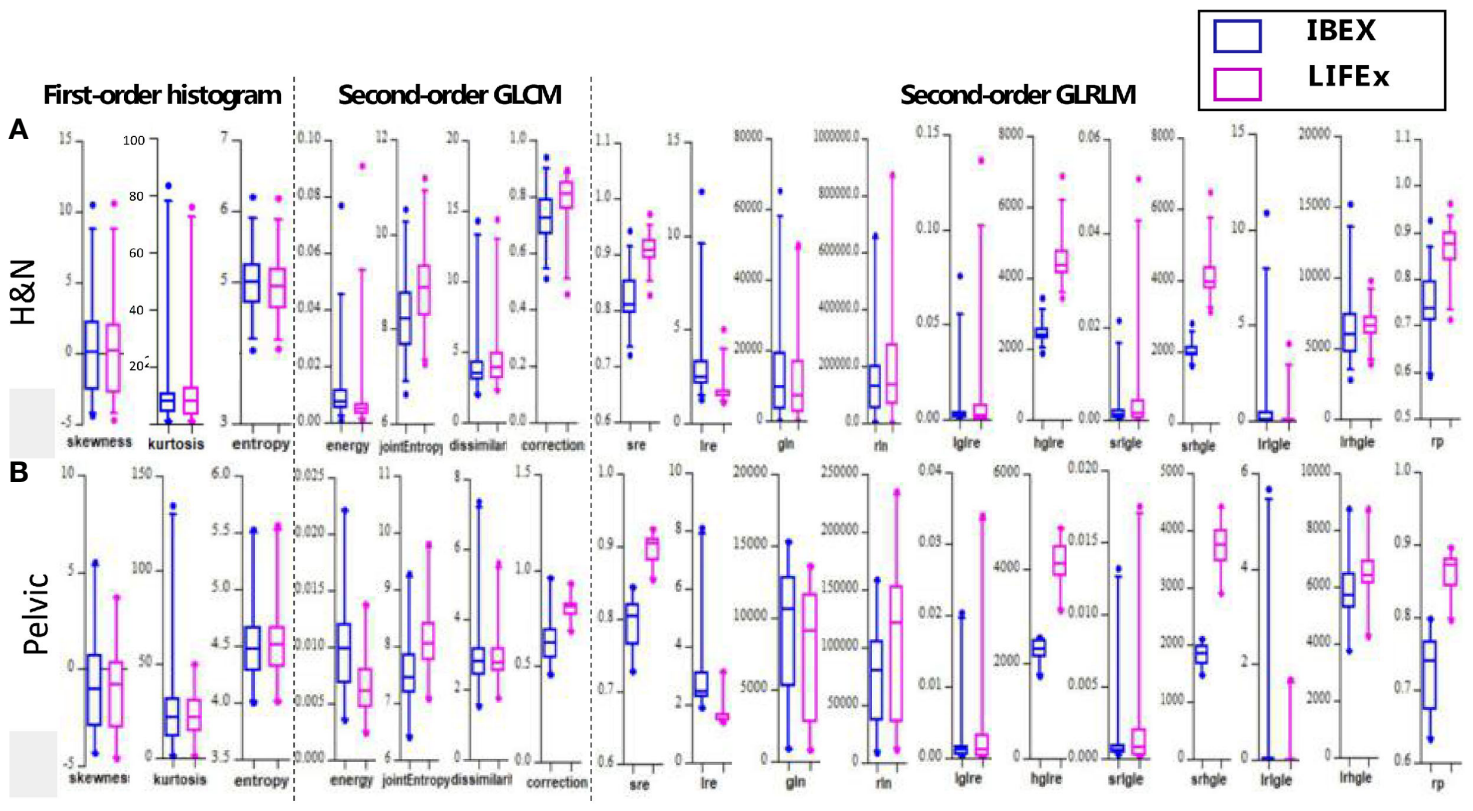
The boxplots showing all values of the features analyzed by the two different softwares for **(A)** H&N and **(B)** pelvic sites, respectively.

Boxplots depicting the distributions of all features between the two software packages are shown in **Figure 3**. It can be seen that the features analyzed by the two different packages were not identical and had large variations especially for second-order texture features.

The site-specific ICCs for differences between packages are shown in **Table 2**. The first-order histogram-based features such as skewness and entropy showed good agreement computed in either of the software packages (ICCs ≥ 0.80). Interestingly, the kurtosis measurements were consistent in H&N patients between the two software tools but not in pelvic cases. Kurtosis is a measure of whether the gray-level intensity histogram is heavy-/light-tailed relative to a normal distribution. The feature itself is sensitive to noise as larger scatter results in greater extremity of deviations (or outliers), thus a higher value of kurtosis. It can be seen that IBEX was more sensitive to scatter-induced kurtosis measurements compared with LIFEx as in pelvic cases. The second-order texture features such as GLCM and GLRLM showed larger distribution variations between software packages compared with first-order histogram-based features. However, the ICCs for GLCM-based features showed good agreement (ICCs ≥ 0.80). This indicated that while systematic biases were introduced due to differences in each of the packages resulting in absolute value differences, the magnitude of these biases was small relative to the feature values themselves. Therefore, the ICCs still reflected good agreement between packages. The GLCM-correction showed poor agreement between packages in both H&N and pelvic groups. The GLRLM-based texture features were overall less consistent computed by the two different software packages compared with GLCM-based features, only showing poor–moderate agreement. However, H&N features tended to show slightly higher ICC values; again, this might be due to less scatter and noise with inherent image setting compared with the pelvic protocol.

**Table 2:** The site-specific ICC values of all features analyzed by the two software packages.

Features	ICCs	
	H&N	Pelvic
First-order histogram-based features		
Skewness	0.949	0.940
Kurtosis	**0.969**	0.298*
Entropy	0.927	0.990
Second-order GLCM-based features		
GLCM-energy	0.966	0.884
GLCM-jointEntropy	0.919	0.983
GLCM-dissimilarity	0.929	0.849
GLCM-correction	**0.264**	**0.561**
Second-order GLRLM-based features		
jointEntropyGLRLM-SRE	0.730	0.623
jointEntropyGLRLM-LRE	0.606	0.530
GLRLM-GLN	0.977	0.973
GLRLM-RLN	0.921	0.859
GLRLM-LGLRE	0.832	0.606
GLRLM-HGLRE	0.664	0.705
GLRLM-SRLGLE	0.681	0.727
GLRLM-SRHGLE	0.645	0.577
GLRLM-LRLGLE	0.628	0.437
GLRLM-LRHGLE	0.842	0.834
GLRLM-RP	0.581	0.552

Bold values: Measurements consistence in H&N patients between the two software tools were different from that in pelvic cases.

*IBEX was more sensitive to scatter-induced kurtosis measurements compared with LIFEx as in pelvic cases.

**Figure 4:**
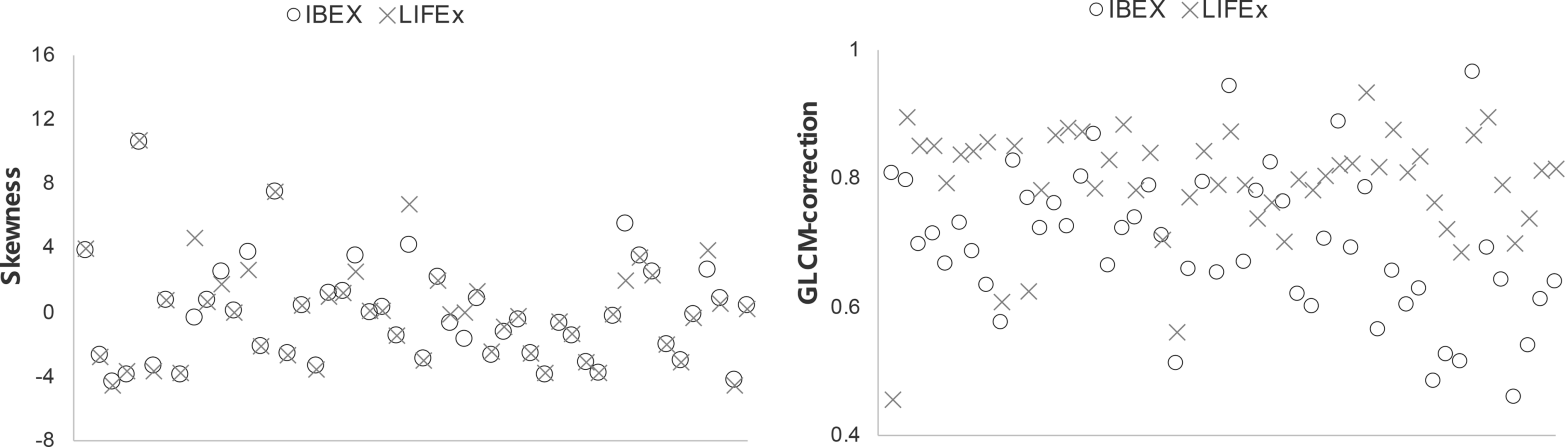
Scatter plots of two selected features showing good and poor agreement among all patients using the two softwares.

Scatter plots of selected features calculated from the ROIs of all patients are shown in **Figure 4**, which demonstrated good and poor agreement between packages, respectively (skewness, 0.947; and GLCM-correction, 0.483). In the scatter plot depicting the feature distribution for skewness, the differences in feature values between packages were small relative to the variations in feature values among patients resulting in an ICC value close to 1, reflecting good agreement. In contrast, in GLCM-correction, the differences in feature values for each patient across packages were large, resulting in significant differences and an ICC value less than 0.5, reflecting poor agreement.

### Repeatability of CBCT-Based Radiomics Features

**Figure 5:**
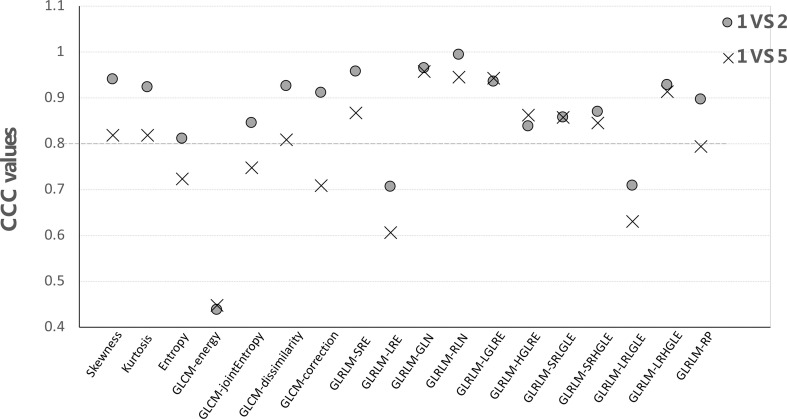
The CCC values of each feature by comparing the first- *vs*. second-day and first- *vs*. fifth-day CBCT.

For each feature group, CCC values were computed by comparing the first *vs*. second and first *vs*. fifth scans for all patients using the IBEX software with results shown in **Figure 5**. As expected, the CCC values computed from the first- *vs*. fifth-day scans were lower than those comparing between the first- *vs*. second-day scans. For example, the CCC of skewness was 0.94 using 2-day apart scans but dropped to 0.82 when comparing the 5-day apart scans. When using a cutoff CCC of 0.80, 15/18 features were reproducible using 2-day apart test–retest dataset, while 11/18 features were reproducible using 5-day apart test–retest dataset. The CCC values of all first-order and second-order GLCM features (except GLCM-energy) were all above 0.80 from the 2-day part test–retest set, indicating they were relatively robust in data extraction. Yet, the CCC values all dropped below the cutoff after 5-day treatment scans, indicating that they might be sensitive in detecting the therapy-induced changes. The GLCM-energy showed the lowest CCC value regardless of which test–retest dataset was used, indicating its non-robustness in radiomics features.

**Figure 6:**
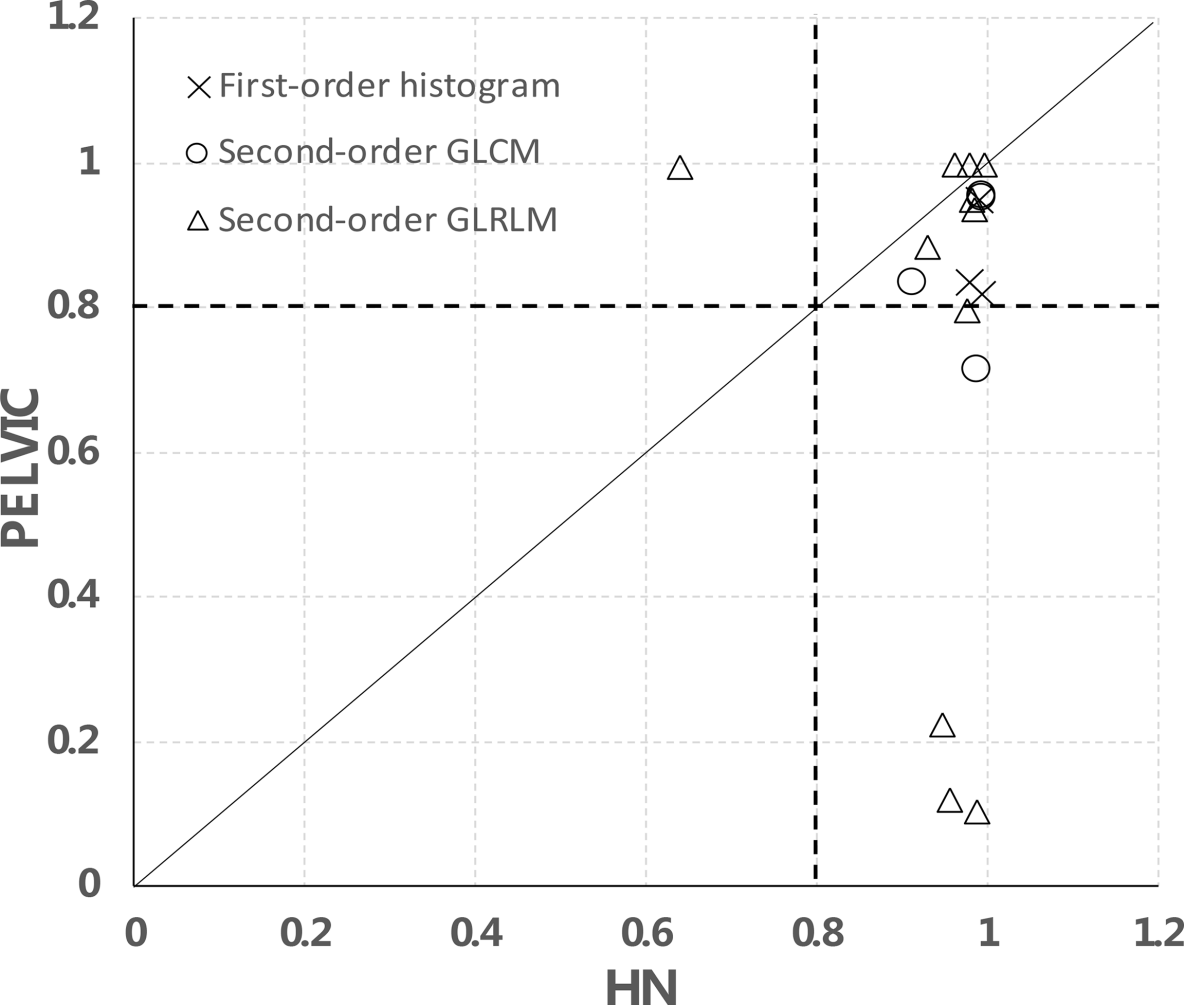
Site-specific CCC values of all analyzed features using 2-day apart test–retest scans.

We further separated the analysis between H&N and pelvic data using the 2-day apart test–retest scans as shown in **Figure 6**. In total, for 15/18 features, the data points are on the right side of the diagonal, meaning that they have a higher CCC in the H&N dataset than in the pelvic dataset. These were 3/3 in the first-order histogram-based features, 4/4 in the GLCM-texture-based features, and 8/11 in the GLRLM-based features, which indicate that the stability of these features may be disease- or image protocol-specific. There were overlaps of stable features in both H&N and pelvic datasets (CCCs ≥ 0.8) for both sites. There were 3/3 first-order histogram-based features (skewness, kurtosis, entropy), 3/4 in GLCM-texture-based features (GLCM-energy, GLCM-jointEntropy, and GLCM-correlation), and 7/11 in GLRLM-based features (SRE, LRE, RLN, HGLRE, SRHGLE, LRHGLE, and RP).

**Figure 7:**
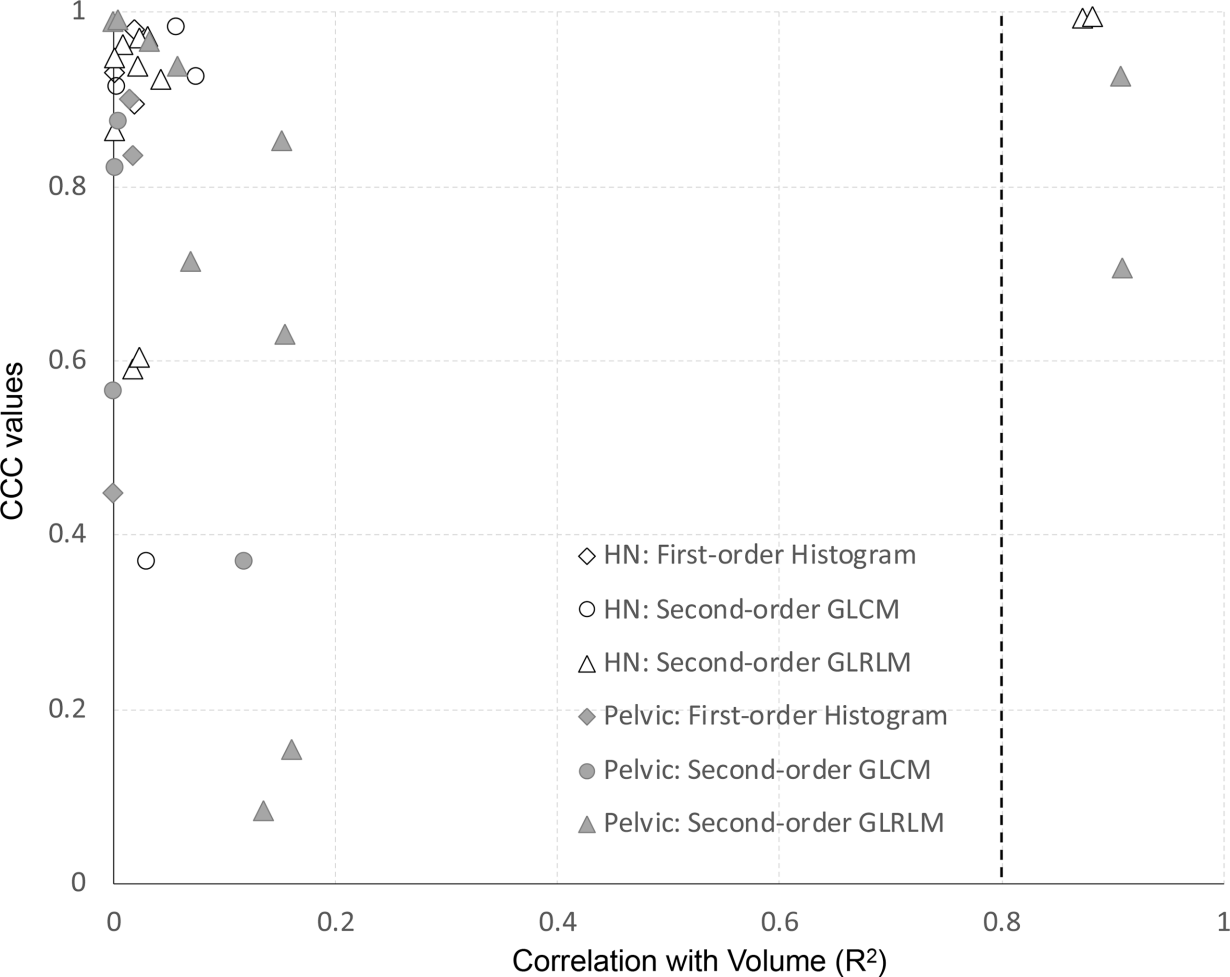
Dependence of feature repeatability on volume separate in H&N and pelvic cases.

For all features of the clinical dataset, we assessed the correlation with volume using the coefficient of determination (*R* ^2^) of a simple linear regression. Features extracted from the first- *vs*. second-day scan were used for this analysis. Results are shown in **Figure 7**. The *y*-axis represents the site-specific repeatability for all features, and the *x*-axis represents the *R* ^2^ of the correlation of volume. All first-order histogram-based and GLCM-texture-based features were not highly correlated with volume. However, there were two features, in both H&N and pelvic cohorts, which showed *R* ^2^ ≥0.8, meaning a high correlation with volume. Both of them were GLRLM features—GLRLM-GLN (*R* ^2^ = 0.87 and 0.91 in H&N and pelvic cases specifically) and GLRLM-RLN (*R* ^2^ = 0.88 and 0.91, respectively).

## Discussion

In many cases of radiotherapy, CBCT is acquired throughout the patient radiation treatment and, thus, is a feasible image modality to detect sequential changes during treatment (1–5). However, before extending its role as an effective biomarker, the stability as well as the reproducibility and repeatability of CBCT-based radiomics features should be assessed. In this study, the reproducibility of CBCT-based radiomics features was assessed using two different softwares on two clinical sites with different acquisition settings, while repeatability was evaluated by comparing scans acquired at different days.

Recently, the stability of radiomics features has gained great attention from the society, and there have been over dozens of studies on related topics (17–22). However, previous works primarily focused on pretreatment diagnostic-level CT or PET images. Fave et al. (22) were one of the very few who tried to investigate the stability of CBCT-related radiomics features. They focused specifically on a test–retest dataset with 10 lung patients scanned twice for 15 min apart. They found that feature repeatability using CBCT was adversely affected by motion. However, there are many other questions that have not been answered, e.g., whether the finding in lung CBCT can be generalized to other disease sites such as H&N and pelvis, where motion is not commonly seen; and whether findings on other modalities, such as CT, can be extended to CBCT. To our best knowledge, limited work has been done to understand the roles of CBCT-based radiomics features. In this study, we assumed the tumor had minimal changes especially during the first 2 days of treatment; in this way, we can evaluate the stability of CBCT-based radiomics features. Our results revealed that most first-order histogram-based parameters on CBCT were reproducible for both disease sites compared with second-order features, with similar results confirmed by previous studies but on other modalities (12, 22–25). Notably, the robust features identified in H&N imaging protocol were not always extendable to pelvic imaging protocol. This might be due to the position of the tumor which is sometimes affected by the filling status of the bladder or rectum. The inherent differences of the image acquisition settings such as larger scatters, higher noise, and larger image resolution also contributed to larger variations as seen in pelvic protocols. Thus, the stability of radiomics features could be disease-/imaging protocol-specific and should be evaluated respectively and carefully.

The agreement of CBCT radiomics features across the two widely used software packages was demonstrated in this study, and variations were observed between the packages for both clinical sites. These sources of variations among packages included differences in image preprocessing, algorithm implementation, and feature-specific parameters. To ensure direct comparison, only those features shared with the same mathematical definitions were selected. Additionally, in LIFEx, features were calculated on the largest cluster of continuous voxels, while in IBEX, whole ROI was used regardless of whether voxels were connected or not. In the current study, only the connected ROI was used for calculation. Moreover, IBEX did not allow non-negative HUs of the CT scans, despite the fact that the lowest HU for a CT scan is −1,014. Thus, HU transformation was applied for feature calculation. Several studies have previously demonstrated that features can vary when calculated in different software platforms (21, 26, 27). The Image Biomarker Standardization Initiative (IBSI) is an international collaboration developed to help standardize radiomics feature calculation in terms of feature definition and nomenclature (28, 29). However, IBSI did not give guidelines for feature calculation settings. To eliminate variation from image preprocessing, no additional and only the default preprocessing was applied. What we found is that many first-order features showed good agreement across packages, with nearly all features differed. All second-order features showed poor–moderate agreement and had large variations when using package-specific default parameters. Therefore, when these radiomics features are used for predictive modeling, computer-aided diagnosis, or image segmentation, for example, the results could greatly differ depending on the software being used. It is unlikely for a single institutional research-oriented work to use different radiomics analysis software packages. However, for multicenter clinical trials and future accreditation work, the reproducibility of radiomics features using different analysis algorithms/packages should be documented and carefully evaluated.

Previously, the repeatability of radiomics features was tested in a “coffee-break” dataset of patients scanned with 15-min intervals (30–32). Due to ethical reasons by introducing an extra image dose, limited patient data can be collected and sometimes phantom measurement has to be used as an alternative (33). However, it is unclear whether stable features measured from the physical phantom or limited data of the patients with “coffee-break” intervals can truly represent the clinical scenario in which the time between scans is in the order of days. On the other hand, daily CBCT is commonly used in the radiation department for most of the patients, thus providing an informative test–retest dataset for radiomics feature repeatability assessment. Especially during the first- and second-day CBCT, patient anatomy changes are relatively minimal, which may allow for radiomics feature stability evaluation. It is also noticed that features from 5-day apart CBCTs were less consistent than the 2-day apart images. It cannot be excluded that in this time period, the tumor changes subclinically and that this change is detected by radiomics. When prognostic information is derived from image features in a radiomics study, one should be aware of changes in a tumor. It is advisable to avoid using features that are not robust in a test–retest study. However, if the dataset with a large time interval is used for test–retest analysis, it would mean that we discard features that are actually informative. Although beyond the scope of the current study, our future work will expand the current finding to broader-scope clinical studies to identify the most reliable and informative radiomics predictors for clinical outcome. Moreover, feature repeatability and its correlation with volume were further assessed in the current study. It is in consensus that from a multi-institutional trial that volume was one of the robust features in the clinical test–retest analysis (34). It was shown that two GLRLM features were highly correlated with volume, and this could partly explain the good repeatability of these features. However, the size dependence could introduce a certain level of uncertainty to extend the work to other studies. As such, we emphasize the importance of a proper test–retest study with a close control on the imaging acquisition protocol, interval of scans, target volume range, etc.

There were some limitations in this study. Several factors could have reduced the robustness of radiomics features. The variability of feature values, however, was compounded by differences in segmentation methods and institution-specific factors, whereas the dependence of the variability in features due to image-specific parameters (e.g., tissue type, imaging modality, and image acquisition) was not discussed. Although in this study we had patients for a test–retest study in a more clinical-oriented scenario compared with previous studies, the dataset was very small to be able to analyze subsets to test these effects. Future studies of images, where predictive performance for the outcome of interest is investigated, taken at different time points during treatment with a multi-institutional trial are necessary to address these considerations. Future studies should be designed to tightly control all aforementioned factors in a radiomics study. Nevertheless, to minimize the risk of using unstable and unreproducible features in a radiomics analysis, it is advisable to perform treatment site-specific and time-, scanner-, and imaging protocol-controlled analyses.

Despite CT/CBCT being the most common image modalities in the radiation oncology world and stringently maintained for patient setup/dose calculation purpose, there is a lack of consensus in terms of calibration process of the image system for additional radiomics (texture) analysis. Previous works primarily focused on understanding the inherent characteristics of CT-based radiomics features using water/solid water phantoms, the Gammex phantom (Sun Nuclear, Melbourne, FL, USA) or other commercially available phantoms designed for CT performance check (33, 35–37). However, those phantoms were originally designed with uniform materials offering HU close to human tissues with minimal internal patterns. These led to recent studies designing radiomics phantoms that consist of different textural compositions. Among them, the most used one was the credence cartridge radiomics (CCR) phantom which consisted of 10 different cartridges with rubber, polyurethane, cork, etc. (38). However, the capability of those materials in recapitulating human tissue in terms of a wide range of radiomics features was not evaluated. Additionally, radiomics feature itself as well as its stabilities can be disease-specific and material-dependent. Moreover, studies to understand CBCT-based radiomics features are even limited, and whether the findings in CT can be translated into CBCT is unknown. We are in the process of designing radiomics phantoms with various compositions to replicate radiomics features of different disease sites. These phantoms can be further used to evaluate feature reproducibility in terms of different material designs with varied acquisition settings and acquisition techniques including CBCT. Further effort can be expanded on understanding not just the variations on acquisition settings but also on which range and what methods can combat these variations.

In summary, the reproducibility and repeatability of CBCT-based radiomics features were assessed and compared for the first time on both H&N and pelvic sites. There were overlaps of stable features in both disease sites, yet the overall stability of radiomics features may be disease-/protocol-specific and a function of time between scans. More investigations are needed to further evaluate the stability of CBCT-based radiomics features before establishing its role as clinical biomarkers.

## Data Availability Statement

The raw data supporting the conclusions of this article will be made available by the authors, without undue reservation.

## Ethics Statement

The studies involving human participants were reviewed and approved by the Ethics Committee of Shanghai Chest Hospital (committee reference number: KS1863). The patients/participants provided their written informed consent to participate in this study.

## Author Contributions

All authors listed have made a substantial, direct, and intellectual contribution to the work and approved it for publication.

## Funding

This work was partially funded by Shanghai Hospital Development Center (Grant No.16CR3056A), the Interdisciplinary Program of Shanghai Jiao Tong University (Grant No. YG2019ZDB2019), and Nurture Projects for Basic Research of Shanghai Chest Hospital (Grant No. 2019YNJCM05).

## Conflict of Interest

The authors declare that the research was conducted in the absence of any commercial or financial relationships that could be construed as a potential conflict of interest.

## Publisher’s Note

All claims expressed in this article are solely those of the authors and do not necessarily represent those of their affiliated organizations, or those of the publisher, the editors and the reviewers. Any product that may be evaluated in this article, or claim that may be made by its manufacturer, is not guaranteed or endorsed by the publisher.
